# Factors influencing menstrual regularity among female workers: a cross-sectional analysis study

**DOI:** 10.1186/s12905-024-03142-8

**Published:** 2024-05-20

**Authors:** Joohee Shim, Seungwoo Han, Jihyun Baek

**Affiliations:** 1 College of Nursing, Yeungnam University College, Daegu, Republic of Korea; 2 Department of Nursing, Kwangju Women’s University, Gwangju, Republic of Korea; 3 College of Nursing, Research Institute of Nursing Science, Jeonbuk National University; Biomedical Research Institute, Jeonbuk National University Hospital, Jeonju, Republic of Korea; 4 College of Nursing, Research Institute of Nursing Science, Jeonbuk National University, 567 Baekje-daero, Deokjin-gu, Jeonju-si, Jeollabuk-do 54896 Republic of Korea

**Keywords:** Menstruation, Menstrual cycle, Women, Working, Obesity, Shift work schedule

## Abstract

**Background:**

Regularity of menstrual cycles is an important indicator of women’s health and fertility, and female workers are exposed to several factors, such as sleep disorders, stress, and shift work, that affect their menstrual regularity. This makes it necessary to comprehensively identify the determinants of menstrual regularity. Therefore, this study identified the factors affecting menstrual regularity among female workers from physiological, psychological, and situational dimensions based on the theory of unpleasant symptoms.

**Methods:**

This was a secondary analysis of the 2010–2012 Korea National Health and Nutrition Examination Survey and utilized the data of 2418 female workers. Based on the theory of unpleasant symptoms, physiological factors included age, age at menarche, childbirth experience, body mass index, and sleep duration. Psychological factors included stress level, depressive mood, and suicidal ideation. Situational factors included education level, household income, consumption of alcohol, engagement in smoking, and work schedule. The χ²-test and hierarchical logistic regression analysis were performed, reflecting the complex sample design.

**Results:**

Age at menarche, childbirth experience, and body mass index among physiological factors and education level and work schedule among situational factors were found to be related to menstrual regularity. A higher risk of menstrual irregularities was found among those who had given birth (versus those who had not), had a high age at menarche (versus those with a low age at menarche), were obese (versus those who had a normal body mass index), had elementary school-level or lesser educational achievements (versus those with college graduate-level or higher educational achievements), and who had a shift work schedule (versus those with a fixed schedule).

**Conclusions:**

Intervention is needed for female workers who have these risk factors, and special attention must be paid to female workers who have a shift work schedule. Additionally, since body mass index can be controlled, intervention concerning body mass index is necessary to reduce menstrual irregularity.

## Background

The most recent Employment and Labor Status data shows that, as of June 2023, there were 12,702,000 female workers in Korea, accounting for approximately 44.1% of the workforce. This number increased by approximately 332,000 compared to the previous year [1]. The rise in dual-income households, elevation of women’s social standing, increase in full-time employment opportunities, and advancements in educational levels have collectively accelerated women’s advancement. Consequently, an increasing number of women are striving for occupational success [2]. With this, the need for societal focus on and health equity of female workers has become increasingly important in Korea. Historically, Korean women’s participation in the workforce has been confined primarily to the service sector. However, there has been a significant change over the last three years, with approximately 800,000 women entering more labor-intensive industries, such as mining and manufacturing [1].

Research has shown that women tend to take more sick leave than men [3]. This discrepancy is primarily attributed to gender-based differences in disease susceptibility, with menstrual regularity being a key contributing factor. Research has also suggested that women’s menstrual cycles are related to various physiological, psychological, chemical, and biological factors [4]. Considering the annual increase in the number of women entering high-risk occupations, identifying the determinants of menstrual regularity among female workers is important not only for improving their health but also for maintaining occupational continuity.

Research on menstrual regularity among Korean female workers has primarily focused on examining isolated relationships within specific populations. For instance, studies have examined the association between irregular menstrual cycles and occupational characteristics [4], the relationship between sleep duration and menstrual irregularity among adolescents [5], and the correlation between lifestyle factors and menstrual irregularities among adolescents [6]. There is a notable lack of studies that explore multidimensional factors while addressing a broader demographic, such as working women.

The theory of unpleasant symptoms (TOUS) is a middle-range theory proposed for integrating diverse information about symptoms [7]. According to this theory, three types of influencing factors—physiological, psychological, and situational factors—interact multidimensionally and shape one’s experience of symptoms. Physiological factors include factors such as physical functional status [7–12], psychological factors include emotional and mood states [9, 10, 12–14] and situational factors comprise factors such as economic status, lifestyle habits, and social environment [4, 10, 12, 13, 15–18]. Investigating menstrual cycles among Korean female workers, considering physiological, psychological, and situational aspects based on the TOUS theory [7], is thus deemed appropriate.

Hence, this study aims to investigate the menstrual regularity among female workers in various occupations such as sales and service, production, and office workers. Unlike some previous studies that examined the menstrual cycle of specific populations such as nurses [15, 17], the uniqueness of this study is that it generalizes demographic characteristics in a more multifaceted and realistic way. Furthermore, previous research [15] has reported an association between the frequency of night shifts and menstrual irregularity. However, considering previous research that found a higher rate of menstrual irregularities among day shift workers compared to night shift workers [19], additional research is needed to explore the relationship between shift work and menstrual cycles. Therefore, in this study, all forms of work schedule other than full-time were considered shift work [19, 20].

**Fig. 1:**
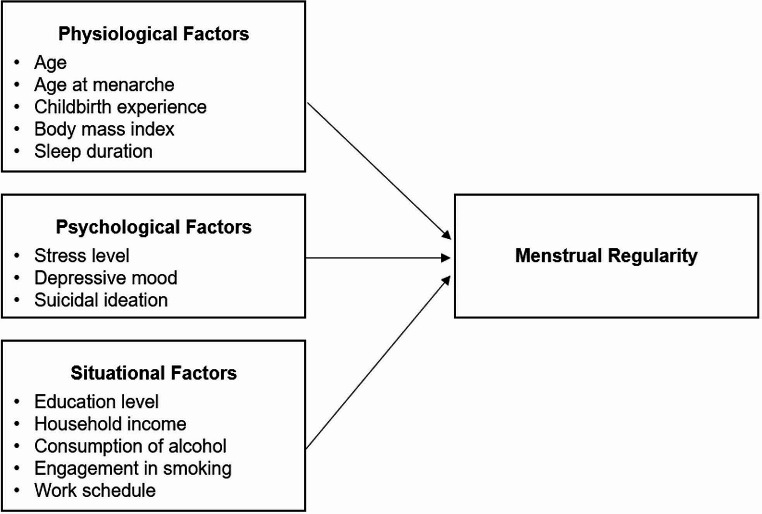
Conceptual framework of the study

Consequently, this study aimed to identify the determinants of menstrual regularity among Korean female workers from physiological, psychological, and situational dimensions based on the TOUS [7]. The findings of this study are expected to provide foundational data for the development of intervention programs for workers experiencing menstrual irregularities. Fig 1 presents the study’s conceptual framework.

## Methods

### Design

This study was a secondary analysis of the 2010–2012 Korea National Health and Nutrition Examination Survey (KNHANES) to identify the factors influencing menstrual regularity among female workers.

### Study population

The KNHANES is a nationally representative, cross-sectional survey conducted by the Korea Centers for Disease Control and Prevention (KCDC). It targets non-institutionalized Korean individuals using multi-stage cluster sampling. Furthermore, it conducts health interviews and examinations regarding participants’ demographic, social, health, and nutritional status [21]. A total of 25,534 people participated in the 2010–2012 KNHANES. We excluded data of men (*n* = 11,616), women aged under 19 years (*n* = 2,780), women who were breastfeeding, those who had attained menopause, or experiencing pregnancy-related amenorrhea (*n* = 6,350), and unemployed women (*n* = 2,114). We also excluded the data of women who had missing data (*n* = 256). Finally, we extracted and utilized the data of 2,418 working women aged 19 or older who experienced menstruation.

### Measures

#### Menstrual regularity

The regularity of the menstrual cycle was investigated through a self-report question. Participants were asked, “Is your menstrual cycle regular at the moment?” The participants could answer this question with “regular” or “irregular.”

#### Physiological characteristics

The physiological characteristics were age (years), age at menarche (years), childbirth experience (yes/no), body mass index (BMI; kg/m^2^), and sleep duration (hours). According to the Asia-Pacific perspective, we categorized BMI as underweight (< 18.5 kg/m^2^), normal (< 25 kg/m^2^), or obese (≥ 25 kg/m^2^) [22]. Sleep duration was measured using a self-report question: “On average, how many hours do you sleep daily?” We categorized the responses as ≤ 5 h, 6–8 h, or ≥ 9 h [12].

#### Psychological characteristics

The psychological characteristics were stress level (high/low stress), depressive mood (yes/no), and suicidal ideation (yes/no). Stress level was evaluated using this question: “How much stress do you usually experience in your daily life?” The response options were very much, a lot, a little, and not very much. We categorized “very much” and “a lot” as “high stress” and “a little” and “not very much” as “low stress.” Depressive mood was assessed using this question: “In the past year, did you feel sad or hopeless to the extent it significantly affected your daily life for two or more consecutive weeks?” Suicidal ideation was assessed using this question: “Have you ever thought of suicide seriously over the past year?” Participants could respond with “yes” or “no” in response to both of the questions.

#### Situational characteristics

The situational characteristics were education level (elementary school or lower, middle school graduate, high school graduate, or college graduate or above), household income (low, middle-low, middle-high, or high), consumption of alcohol (yes/no), engagement in smoking (yes/no), and work schedule (fixed/shift). Household income was self-reported and divided into quartiles. If one had completely abstained from alcohol consumption in the past year, their response to alcohol consumption was categorized as “no.” Otherwise, participants’ responses were categorized as “yes.” For those who were engaged in smoking at the time, their response for engagement in smoking was categorized as “yes.” For those who used to smoke in the past or were non-smokers, their response was categorized as “no.” The work schedule was assessed using this question: “Do you work primarily during the daytime (from 6 AM to 6 PM), or do you work at a different time?” Participants who responded with “Yes, I primarily work during the daytime” were categorized as having a fixed work schedule. Those who indicated other periods (such as night shifts and rotating shifts) were categorized as having a shift work schedule [4].

### Data analysis

To obtain unbiased estimation results that represent the entire population of South Korea, we employed a complex sample analysis method that considered the integrated weights, strata, and clusters suggested in the guidelines of the KCDC. The characteristics of the study population were presented as mean ± standard error for continuous variables and frequency and weight% for categorical variables. We identified differences in physiological, psychological, and situational characteristics based on menstrual regularity using t-tests for continuous variables and the Rao-Scott χ^2^ test for categorical variables. Factors influencing menstrual regularity were determined using hierarchical logistic regression analysis. We incorporated physiological characteristics in Model 1, added psychological characteristics in Model 2, and included situational characteristics in Model 3. The significance level was set at *p* < .05. All statistical analyses were performed using the SAS 9.4 (SAS Institute, Cary, NC, USA).

### Ethical considerations

This study was approved by the ethics committee of the Jeonbuk National University affiliated with the corresponding author (JBNU 2023-09-014), and all procedures were conducted per the ethical standards of the 1964 Declaration of Helsinki. We downloaded data excluding participants’ identifiable information from the KNHANES website (https://knhanes.kdca.go.kr). All participants provided written informed consent to participate in the KNHANES.

## Results

### Physiological, psychological, and situational characteristics and the prevalence of menstrual irregularity

Table 1 presents data on the physiological, psychological, and situational characteristics of and menstrual regularity in the study population. Regarding physiological characteristics, the mean age of the study population was 35.58 ± 0.25 years, and the mean age at menarche was 13.87 ± 0.04 years. In the study population, 60.4% had experienced childbirth, 9.5% were underweight, and 21.2% were obese. The average daily sleep duration was ≤ 5 h for 10.2% of the study population and ≥ 9 h for 7.1%. Regarding psychological characteristics, 37.0% of the study population reported experiencing high levels of stress, 14.1% felt depressed, and 16.7% had suicidal ideation. Regarding situational characteristics, 91% of the study population had a high school graduate-level or higher education achievements. The household income level was middle-high for 33.8% of the study population and high for 33.6%. Additionally, 89.7% reported consuming alcohol, 8.9% reported engaging in smoking, and 22.1% had a shift work schedule. Finally, 14.3% of the study population had irregular menstrual cycles.

**Table 1:** Physiological, psychological, and situational characteristics and menstrual regularity (*N* = 2,418)

Variable	Categories	*N* (Weighted %) or Mean ± SE
**Physiological characteristics**		
Age (years)		35.58 ± 0.25
Age at menarche (years)		13.87 ± 0.04
Childbirth experience	Yes	1574 (60.4)
	No	844 (39.6)
BMI (kg/m^2^)	Underweight	215 (9.5)
	Normal	1695 (69.3)
	Obese	508 (21.2)
Sleep duration (hours)	≤ 5	234 (10.2)
	6–8	2029 (82.7)
	≥ 9	155 (7.1)
**Psychological characteristics**		
Stress level	High stress	871 (37.0)
	Low stress	1547 (63.0)
Depressive mood	Yes	327 (14.1)
	No	2091 (85.9)
Suicidal ideation	Yes	368 (16.7)
	No	2050 (83.3)
**Situational characteristics**		
Education level	Less than elementary school	84 (3.4)
	Middle school graduate	151 (6.6)
	High school graduate	1041 (46.0)
	College graduate or above	1142 (44.0)
Household income	Low	142 (6.7)
	Middle-low	570 (26.0)
	Middle-high	810 (33.8)
	High	896 (33.6)
Consumption of alcohol	Yes	2157 (89.7)
	No	261 (10.3)
Engagement in smoking	Yes	179 (8.9)
	No	2239 (91.1)
Work schedule	Fixed	1919 (77.9)
	Shift	499 (22.1)
**Menstrual regularity**	Regular	2084 (85.7)
	Irregular	334 (14.3)

BMI = body mass index, SE = standard error

### Differences in physiological, psychological, and situational characteristics based on menstrual regularity

Table 2 presents the results of identifying the differences in physiological, psychological, and situational characteristics based on menstrual regularity. Women with irregular menstrual cycles were found to be more likely to have a higher age at menarche (F = 6.53, *p* = .011), be obese (χ² = 11.80, *p* = .003), and sleep for ≤ 5 h on average daily (χ² = 6.83, *p* = .033). Among psychological characteristics, menstrual irregularity was associated with stress (χ² = 5.88, *p* = .015), depressive mood (χ² = 7.94, *p* = .005), and suicidal ideation (χ² = 9.12, *p* = .003). Among situational characteristics, education level was related to menstrual regularity, with individuals with elementary school-level or lower educational achievements experiencing more irregular menstrual cycles than regular cycles (χ² = 23.55, *p* < .001). Furthermore, menstrual irregularity was observed in those who engaged in smoking (χ² = 4.00, *p* = .046) and those with a shift work schedule (χ² = 9.12, *p* = .003).

**Table 2:** Differences in physiological, psychological, and situational characteristics by menstrual regularity (*N* = 2,418)

Variable	Categories	Regular	Irregular	F or χ²	*p*
		*N* (Weighted %) or Mean ± SE			
**Physiological characteristics**					
Age (years)		35.55 ± 0.26	35.7 6 ± 0.76	0.07	0.791
Age at menarche (years)		13.82 ± 0.05	14.20 ± 0.14	6.53	0.011
Childbirth experience	Yes	1364 (60.9)	210 (57.3)	1.01	0.316
	No	720 (39.1)	124 (42.7)		
BMI (kg/m^2^)	Underweight	188 (9.7)	27 (8.3)	11.80	0.003
	Normal	1486 (70.5)	209 (61.9)		
	Obese	410 (19.8)	98 (29.8)		
Sleep duration (hours)	≤ 5	187 (9.5)	47 (14.2)	6.83	0.033
	6–8	1768 (83.6)	261 (77.2)		
	≥ 9	129 (6.9)	26 (8.5)		
**Psychological characteristics**					
Stress level	High stress	728 (35.9)	143 (43.4)	5.88	0.015
	Low stress	1356 (64.1)	191 (56.6)		
Depressive mood	Yes	258 (13.1)	69 (19.7)	7.94	0.005
	No	1826 (86.9)	265 (80.3)		
Suicidal ideation	Yes	294 (15.7)	74 (23.0)	9.12	0.003
	No	1790 (84.3)	260 (77.0)		
**Situational characteristics**					
Education level	Less than elementary school	59 (2.8)	25 (7.2)	23.55	< 0.001
	Middle school graduate	113 (6.0)	38 (10.1)		
	High school graduate	896 (45.7)	145 (47.7)		
	College graduate or above	1016 (45.5)	126 (34.9)		
Household income	Low	123 (6.7)	19 (6.6)	2.15	0.543
	Middle-low	497 (26.3)	73 (23.9)		
	Middle-high	700 (34.1)	110 (31.8)		
	High	764 (32.9)	132 (37.7)		
Consumption of alcohol	Yes	1859 (89.7)	298 (89.8)	0.01	0.942
	No	225 (10.3)	36 (10.2)		
Engagement in smoking	Yes	144 (8.3)	35 (12.4)	4.00	0.046
	No	1940 (91.7)	299 (87.6)		
Work schedule	Fixed	1676 (79.0)	243 (70.8)	9.12	0.003
	Shift	408 (21.0)	91 (29.2)		

BMI = body mass index, SE = standard error

### Factors influencing menstrual regularity

Table 3 presents the results of the hierarchical logistic regression analysis. In Models 1 and 2, women who had a high age at menarche, had experienced childbirth, were obese, and slept for ≤ 5 h on average daily had higher odds ratios (ORs) of menstrual cycle irregularity than those who had a lower age at menarche, had not experienced childbirth, had a normal BMI, and slept for 6–8 h. In Model 3, high age at menarche was associated with an increased risk of menstrual irregularity (OR = 1.123, 95% CI [confidence interval] = 1.026–1.229). The risk of menstrual irregularity was higher among those who were obese than those who had a normal BMI (OR = 1.751, 95% CI = 1.234–2.485). Those who had elementary school-level or lower educational achievements were at higher risk of menstrual irregularity than those who had college graduate-level or higher education achievements (OR = 3.524, 95% CI = 1.618–7.679). Furthermore, those who had a shift work schedule were at a higher risk of menstrual irregularities than those who had a fixed schedule (OR = 1.484, 95% CI = 1.091–2.016). However, the association between sleep duration and menstrual regularity disappeared in all adjusted models.

**Table 3:** Factors influencing menstrual regularity (*N* = 2,418)

	Model 1				Model 2				Model 3			
	OR	95% CI		*p*	OR	95% CI		*p*	OR	95% CI		*p*
**Physiological characteristics**												
Age (years)	1.005	0.981	1.031	0.680	1.008	0.983	1.032	0.546	0.995	0.969	1.022	0.720
Age at menarche (years)	1.154	1.060	1.256	0.001	1.147	1.054	1.249	0.002	1.123	1.026	1.229	0.012
Childbirth experience (ref. = No)												
Yes	0.609	0.402	0.922	0.019	0.615	0.409	0.925	0.020	0.654	0.427	1.001	0.050
BMI (kg/m^2^) (ref. = Normal)												
Underweight	0.870	0.511	1.482	0.608	0.881	0.512	1.518	0.648	0.831	0.476	1.451	0.515
Obese	1.801	1.283	2.528	0.001	1.772	1.258	2.496	0.001	1.751	1.234	2.485	0.002
Sleep duration (hours) (ref. = 6–8)												
≤ 5	1.595	1.068	2.380	0.023	1.498	1.005	2.233	0.047	1.351	0.899	2.031	0.148
≥ 9	1.248	0.779	2.000	0.357	1.220	0.758	1.961	0.412	1.092	0.675	1.769	0.719
**Psychological characteristics**												
Stress level (ref. = Low stress)												
High stress					1.155	0.873	1.529	0.311	1.172	0.882	1.559	0.273
Depressive mood (ref. = No)												
Yes					1.283	0.864	1.905	0.217	1.241	0.824	1.867	0.301
Suicidal ideation (ref. = No)												
Yes					1.295	0.905	1.854	0.157	1.252	0.866	1.811	0.232
**Situational characteristics**												
Education level (ref. = College graduate or above)												
Less than elementary school									3.524	1.618	7.679	0.019
Middle school graduate									2.295	1.303	4.043	0.277
High school graduate									1.457	1.049	2.023	0.104
Household income (ref. = High)												
Low									0.516	0.264	1.009	0.242
Middle-low									0.609	0.415	0.894	0.415
Middle-high									0.700	0.504	0.972	0.866
Consumption of alcohol (ref. = No)												
Yes									0.906	0.579	1.417	0.664
Engagement in smoking (ref. = No)												
Yes									1.242	0.764	2.020	0.382
Work schedule (ref. = Fixed)												
Shift									1.484	1.091	2.016	0.012

OR = odds ratio, CI = confidence interval, ref.=reference

## Discussion

This study identified the determinants of menstrual regularity among female workers by sourcing and analyzing data from the 2010–2012 KNHANES to improve female workers’ health-related quality of life and provide effective guidelines for preventing and managing menstrual irregularity. The hierarchical logistic regression analysis showed that age at menarche, obesity, education level, and a shift work schedule are associated with irregular menstrual cycles.

We found that a high age at menarche is associated with irregular menstrual cycles. This finding is consistent with that of previous studies, including one that involved female university students in Poland [8], one that involved Taiwanese college nursing students [23], and one that involved Korean nurses [15]. However, our finding is contradictory to studies that found an association between early age at menarche and menstrual cycle disorders [24, 25] and the inconsistent results on the relationship between age at menarche and menstrual cycle regularity. The relationship between age at menarche and menstrual cycle pattern is mostly attributable to hormonal factors, but it may also be an indirect effect of differences in adiposity. Body weight and adiposity are among the hypothesized triggers of menarche [26], and age at menarche is inversely related to adiposity, with irregular menstrual cycles being more prevalent in women with greater amounts of adipose tissue [8, 27]. but the BMI at the time of menarche was unknown. This limited us from explaining their relationship. Understanding the relationship between age at menarche and the pattern of menstrual cycles can contribute to explaining the etiology of menstrual disorders and gynecological diseases. However, few studies have explored age at menarche and menstrual irregularities, and the mechanisms that may explain them have not been clearly identified. Therefore, future studies should examine women of different countries and ages and investigate BMI at menarche.

We found that obesity is associated with irregular menstrual cycles. This finding is consistent with many previous studies on menstruation and obesity. Obesity in adulthood has been found to be associated with irregular menstrual cycles. Wei et al.’s [28] study involving Australian women aged 26–36 found that women with a higher BMI are more likely to have irregular menstrual cycles. More specifically, women with a BMI of 30 kg/m^2^ or more were twice as likely to have irregular menstrual cycles as women with a normal BMI. Hartz et al. [29] found that higher BMI is associated with the absence of menstruation and irregular menstruation. Castillo-Martínez et al. [30] examined the characteristics of the menstrual cycle among obese women in Mexico by categorizing them into five classes of relative weight. They found that obesity class is independently associated with irregular menstrual cycles, with higher obesity classes associated with higher odds of menstrual cycle disorders. Hartz et al. [29] found that the prevalence of irregular menstrual cycles is 8.4% among obese women, while Castillo-Martínez et al. [30] found the prevalence to be 34.4%. Chang et al.’s [23] study involving female students aged 18–25 in Taiwan found that women with a BMI greater than 27 kg/m^2^ have an 18.48 times higher risk of developing irregular menstrual cycles than women with a BMI between 18.5 and 23.9 kg/m^2^. Bull et al. [31] found that the average change in the menstrual cycle length per person is 0.4 days or 14% higher among women with a BMI of 35 kg/m^2^ or higher than those with a normal BMI. Song et al. [15] found that women with a BMI of 25 kg/m^2^ or higher have longer cycles. Chang et al. [23] found that obese students with a BMI of 27 kg/m^2^ are at a higher risk of having long cycles, which is associated with increased follicular phase and decreased luteal phase lengths in heavier women. Obesity and menstrual irregularities have metabolic and neuroendocrine mechanisms. Owing to lower levels of sex hormone-binding globulin (SHBG) and higher levels of testosterone, fasting insulin, and free androgen index, women with higher BMI are more prone to menstrual irregularities [28, 32]. Additionally, it has been suggested that menstrual cycle disorders in obese women may be related to disorders of estrogen metabolism, changes in the concentration of SHBG, hyperinsulinemia, and changes in leptin levels [33]. The proportion of women with obesity-induced menstrual cycle problems is expected to increase given that the number of obese people worldwide is increasing steadily. Accordingly, it is necessary to provide health information and education about the importance of weight management and obesity being linked to women’s reproductive health problems.

However, in a study examining the prevalence of menstrual cycle disorders in physically active women with a BMI of less than 18.5 kg/m^2^, half of the underweight women reported menstrual cycle disorders [34]. Additionally, a study conducted on medical students aged 18–25 years reported a relationship between low BMI and irregular menstrual cycles [35], and a study conducted on female college students aged 18–26 years also reported a relationship between low body weight and irregular menstrual cycles [36]. In this study, the relationship between being underweight and menstrual cycles was not found. First, it may be related to the small number of underweight people investigated to show a relationship. It may also be related to the subjects. Most of the participants in studies that reported a relationship between being underweight and menstrual cycles were female college students and adolescents [35–37]. The poor nutritional status of young women appears to have a greater impact on the menstrual cycle, which may be related to the different age groups in this study. Since weight control needs to be effectively managed in daily life, education, and management of exercise and eating habits are required.

We found that education level is associated with irregular menstrual cycles, and women with elementary school-level or lower educational achievements are more likely to have irregular menstrual cycles. This may be related, firstly, to the inverse relationship between education level and obesity. Low education level is associated with obesity [38–41], and obesity increases the risk of having irregular menstrual cycles. Obesity can promote irregular menstruation among women with low socioeconomic status [18]. This study confirms this possibility by showing that obesity determines irregular menstrual cycles, and it can be thought that low education levels, obesity, and irregular menstrual cycles influence each other. Second, the finding can be explained by the fact that a low education level is related to sleep disorders and stress. Research has shown that the incidence of sleep problems is higher among less educated employees and temporary workers [42], and sleep disorders and stress can affect the endocrine system and menstruation [43]. In other words, the lower the level of education, the more temporary and unstable employment becomes. This causes anxiety and depression, which, in turn, leads to sleep disorders and stress [44]. Studies have shown that sleep disorders and stress can increase the risk of irregular menstrual cycles [44, 45], and our study supports this finding by showing that five or fewer hours of sleep and stress increase the risk of irregular menstrual cycles. Finally, education is positively associated with long-term health, as it strengthens social and mental resources [18, 44]. Therefore, it appears that the lower the level of education, the higher the incidence of menstruation-related health problems.

A shift work schedule, which can disrupt the circadian rhythm’s normal functioning, is considered one of the factors contributing to changes in the menstrual cycle. We found that the risk of menstrual irregularity is higher among women with a shift work schedule than those with a fixed work schedule. This finding supports that of previous studies. Lawson et al. [46] examined American women aged 28–45 and found that the probability of having irregular menstrual cycles is 1.23 times higher among those who work in rotating shifts for more than 20 months than those who do not. Wang et al. [17] conducted a cohort study to determine the characteristics of rotating shift work and menstruation among Chinese nurses. The proportion of nurses with irregular menstrual cycles was significantly higher in the shift work group, and the frequency of night work was the only risk factor related to the cycle. Hu et al. [47] meta-analyzed the relationship between shift work and menstruation characteristics and found that the risk of irregular menstruation is 1.30 times higher among shift workers. Circadian rhythms are circadian biological oscillations that follow a 24-hour rhythm [48, 49]. However, irregular work schedules can disrupt the circadian rhythm and change the sleep-wake cycle, which can change the physiological cycle and cause sleep disorders and stress [43, 46]. Moreover, working at night exposes one to light and noise, which may disturb physiological parameters and lead to menstrual cycle disorders [49, 50]. This study also found that irregular menstrual cycles are related to five or fewer hours of sleep and high stress. Therefore, it appears that shift work schedules, sleep duration, and stress interact organically and affect the menstrual cycle. Additionally, the menstrual cycle is defined by the circulatory pattern of reproductive hormones, and sleep appears to inhibit pituitary luteinizing hormone (LH) secretion [51, 52]. Changes in sleep-wake patterns in shift workers can alter LH secretion, thereby changing the length and regularity of the menstrual cycle [46]. Melatonin regulates sleep mechanisms [26, 53, 54] and plays an important role in regulating reproductive physiology. It has been reported that the menstrual cycle is related to fluctuations in melatonin production, but the relationship between melatonin and reproductive hormones among humans has not been clearly revealed [55]. Therefore, although this study and previous studies have shown that shift work and menstrual irregularities are related, the explanation of the relationship and mechanism is limited. Thus, additional research is required in this regard. Additionally, it can be seen that shift work, sleep, and stress are interconnected and affect menstrual irregularity; thus, education on sleep and stress management methods is necessary when intervening with irregular menstrual cycles in shift workers.

In this study, it was observed that a shorter duration of sleep was associated with a higher risk of menstrual irregularity when considering only physiological and psychological characteristics. However, the statistical significance disappeared when situational characteristics such as shift work were added. Previous studies [9, 10] examining the relationship between short sleep duration and menstrual irregularity have not considered for shift work status, as they focused on undergraduate students and community-based samples. Consequently, the relationship between sleep duration and menstrual irregularity observed in these previous studies [9, 10] could have been influenced by the lack of consideration for shift work status. The finding in this study that the risk of menstrual irregularity remains high among shift workers regardless of sleep duration highlights the importance of recognizing the adverse effects of circadian rhythm disruption rather than just sleep deprivation. Therefore, interventions aimed at minimizing circadian rhythm disruption, such as optimizing work schedule patterns [56] and implementing light therapy [57], may be necessary to alleviate the adverse effects of shift work on menstrual cycles.

The average age of participants in this study was 35.58 years old, and no association was found between age and menstrual irregularity. This aligns with previous research targeting healthcare worker of similar age groups (average age of 36.19 years) [58]. However, it contrasts with a study focusing on nurses (average age of 30.83 years), where younger age was reported to be associated with menstrual cycle irregularities [15]. Given that various environmental factors such as shift work and work intensity may influence menstrual regularity, the relationship between age and menstrual irregularity may vary depending on the occupation [58]. Therefore, in future research, it is deemed necessary to further stratify occupational groups in large-scale survey studies to verify the effects of age on menstrual regularity.

The results of this study showed that stress level and depressive mood were significantly associated with menstrual irregularities in univariate analysis, but not in multivariate analysis. This contrasts with previous studies [9, 10]. This discrepancy might be owing to differences in the study population. Previous studies focused on university students or community- based sample [9, 10]. There may be differences in the types, frequencies, and degrees of stress or depression depending on the population. Additionally, measuring stress level and depressive mood with only a single-item each in this study might have made it difficult to capture the multidimensional aspects of these variables. The KNHNES, a nationally representative epidemiological survey, measures stress level and depressive mood with a single-item, and these results have been utilized in various previous studies [59, 60]. Additionally, it has been reported that a single-item measure of self-rated mental health is associated with multi-item measures of self-rated health and is considered reliable [61]. Nevertheless, using a single-item measure instead of valid questionnaires to assess stress levels and depressive mood may lack reliability and validity.

This study identified the factors associated with irregular menstrual cycles using data from the KNHANES, which is representative of Korea. However, this study has several limitations. Firstly, the data used in this study are from 2010 to 2012, which means they are relatively outdated. There could have been important changes in menstrual cycles from the time of data collection to the present. Especially considering the research on the relationship between the COVID-19 pandemic and menstrual irregularities, some previous studies have reported changes in menstrual cycles owing to COVID-19 [62–64], while others have found no such effects [64, 65]. Although the relationship between COVID-19 and menstrual irregularities remains contentious, it seems important to reexamine factors related to menstrual irregularities among female workers in the post-pandemic era. Second, since it is a survey there is a difference depending on how respondents accept the question. The response results may vary depending on the timing of the respondent’s response. Third, it was difficult to determine the causal relationship and mechanism between menstrual cycle regularity and related factors because we used cross-sectional survey data. Finally, even though we used national data representing Korea, there are limitations in applying the results to all women in Korea.

## Conclusions

The menstrual cycle is an important indicator of women’s health, which makes it necessary to identify the determinants of irregular menstruation. This study shows that age at menarche, obesity, education level, and shift work are associated with irregular menstrual cycles. Despite its limitations, this study is significant because it revealed the determinants of menstrual cycle regularity using representative data and provided basic data for the prevention and treatment of women’s reproductive health problems. Its results suggest that people must recognize that various factors are organically connected and affect irregular menstrual cycles. Thus, multidimensional prevention and treatment programs must be devised.

## Data Availability

The datasets analyzed during the current study are available from the corresponding author upon reasonable request.

## Abbreviations

BMI: Body mass index
CI: Confidence interval
KCDC: Korea Centers for Disease Control and Prevention
KNHANES: Korea National Health and Nutrition Examination Survey
LH: Luteinizing hormone
OR: Odds ratio
SHBG: Sex hormone-binding globulin
TOUS: Theory of unpleasant symptoms
